# Editorial for the Special Issue on “Frontiers of Ultra-Precision Machining”

**DOI:** 10.3390/mi13020220

**Published:** 2022-01-29

**Authors:** Jiang Guo, Chunjin Wang, Chenwei Kang

**Affiliations:** 1 School of Mechanical Engineering, Dalian University of Technology, Dalian 116024, China; 2 State Key Laboratory of Ultra-precision Machining Technology, Department of Industrial and Systems Engineering (ISE), the Hong Kong Polytechnic University (PolyU), Hong Kong 999077, China; 3 State Key Laboratory for Manufacturing Systems Engineering, School of Mechanical Engineering, Xi’an Jiaotong University, Xi’an 710049, China

Ultra-precision machining is a multi-disciplinary research area that is an important branch of manufacturing technology. It targets achieving ultra-precision form or surface roughness accuracy, forming the backbone and support of today’s innovative technology industries in aerospace, semiconductors, optics, telecommunications, energy, etc. The increasing demand for components with ultra-precision accuracy has stimulated the development of ultra-precision machining technology in recent decades. Accordingly, this special issue showcases 17 research papers which focus on the frontiers of ultra-precision machining, including ultra-precision machining processes, process simulation and modelling, process optimization, the development of novel machining tools and processes, and surface integrity characterization. 

1. Process simulation and modelling: Yuan et al. [1] presented a dynamic model of the cutting system for the characterization of surface topography variation in ultra-precision tool servo-based diamond cutting of a microlens array considering the tool-work vibration as an underdamped vibration. Du et al. [2] studied the ion beam sputtering process for single crystal aluminum with different crystallographic orientations by the molecular dynamics method, and the mechanism of morphology evolution of aluminum were revealed. Fu et al. [3] presented a piezoelectric hysteresis modeling method based on a generalized Bouc–Wen model, which can describe the piezoelectric hysteresis characteristics of the three axial subsystems of the three-dimensional elliptical vibration which effectively aided the cutting system and ensured higher modeling and fitting accuracy. Tian et al. [4] proposed the Coupled Eulerian–Lagrangian (CEL) method to simulate the high-shear low-pressure grinding process. Zhang et al. [5] established a two-phase flow field model based on the RANS k-ε turbulence mode to analyze the influence of vibration on the process of electrochemical machining, which is suitable for narrow flow field and high flow velocity.

2. Process optimization: Wang et al. [6] proposed a reasonable elementary approximation algorithm of dwell time on the basis of the theoretical requirement of a removal function in the subaperture polishing and single-peak rotational symmetry character of its practical distribution, which has obvious advantages for improving calculation efficiency and flatness and is of great significance for the efficient computation of large-aperture optical polishing. Yan et al. [7] proposed a new calculation method for the height of surface residual materials in ultra-precision grinding of Nano-ZrO2, and established the prediction model of the three-dimensional roughness *S*a and *S*q by using this calculation method. Rashedul et al. [8] investigated the influence of different electrode materials experimentally, namely titanium alloy (TC4), stainless steel (SS304), brass, and copper–tungsten (CuW) alloys (W70Cu30, W80Cu20, W90Cu10), on electrodes’ electrical properties, aiming to select an appropriate electrode in the ECDM process. Zhao et al. [9] studied the critical machining parameters which affect the surface generation and surface quality in the machining of polar microstructures, so as to obtain optimized machining parameters and determine optimized cutting strategy for polar microstructures. Huang et al. [10] studied the self-aligning flanges based on piezoelectric actuators, and the average eccentricity value in the experiments decreased by 74%. Qiao et al. [11] investigated the optimum vertex angle and parameters for the preparation of atom probe tomography (APT) specimen, and the double interdiffusion relationship of the multilayer films was successfully observed by the local electrode APT.

3. Development of novel machining tools and processes: Fan et al. [12] developed an electrorheological (ER) polishing tool with an annular integrated electrode, and six influencing factors of ER polishing were analyzed experimentally. Jin et al. [13] presented a high-accuracy and high-efficiency surface topography manufacturing method for continuous phase plate, which demonstrates the potential of the atmospheric pressure plasma jet approach for the manufacturing of complex surface topographies. Zhou et al. [14] analyzed the machined surface morphology and cutting force in different lubricant machining environments, and the results indicated that the minimum quantity lubrication machining oil can suppress the formation of hard particles to improve the machining quality.

4. Surface integrity characterization: Deng et al. [15] carried out a study on the repair of fused silica damage using the magnetorheological removing method, and the repairing rate of small-scale damage was up to 90.4%. Yang et al. [16] designed experiments to investigate the influence of machining factors on subsurface damage, in order to reduce the subsurface damage depth generated during the grinding process by adjusting the process parameters. Guo et al. [17] proposed a new measurement method in order to accurately obtain the wall thickness of thin-walled spherical shell parts.

We wish to thank all authors who submitted their papers to this Special Issue. We would also like to acknowledge all the reviewers for dedicating their time to provide careful and timely reviews to ensure the quality of this Special Issue.
